# Assessment of plant growth promoting bacteria strains on growth, yield and quality of sweet corn

**DOI:** 10.1038/s41598-022-16044-2

**Published:** 2022-07-08

**Authors:** Nikolaos Katsenios, Varvara Andreou, Panagiotis Sparangis, Nikola Djordjevic, Marianna Giannoglou, Sofia Chanioti, Christoforos-Nikitas Kasimatis, Ioanna Kakabouki, Dimitriοs Leonidakis, Nicholaos Danalatos, George Katsaros, Aspasia Efthimiadou

**Affiliations:** 1 Department of Soil Science of Athens, Institute of Soil and Water Resources, Hellenic Agricultural Organization - Demeter, 14123 LycovrissiAttica, Greece; 2 Institute of Technology of Agricultural Products, Hellenic Agricultural Organization-DEMETER, Lykovrissi, 14123 Attica, Greece; 3 Agrounik d.o.o., Milana Uzelca 11, 11080 Belgrade-Zemun, Serbia; 4 Department of Informatics, University of Piraeus, Karaoli & Dimitriou 80, 18534 Piraeus, Greece; 5 Laboratory of Agronomy, Department of Crop Science, Agricultural University of Athens, 11855 Athens, Greece; 6 Laboratory of Agronomy and Applied Crop Physiology, Department of Agriculture Crop Production and Rural Environment, Fytokou St., University of Thessaly, 38446 Volos, Greece

**Keywords:** Microbiology, Environmental sciences

## Abstract

The use of plant growth promoting bacteria (PGPB) is increasingly gaining acceptance from all the stakeholders of the agricultural production. Different strains of PGPB species had been found to have a vast variety of mechanisms of action, while at the same time, affect differently a variety of crops. This study investigated the effectiveness of ten PGPB strains, on sweet corn cultivation under Mediterranean soil and climatic conditions. A field experiment that followed a completely randomized design was conducted at the region of Attica at Oropos. The results indicated that *B. mojavensis* increased yield by 16%, *B. subtilis* by 13.8%, *B. pumilus* by 11.8% and *B. pseudomycoides* by 9.8% compared to control. In addition, the harvested grains of the plants treated with *B. mojavensis*, *B. subtilis* and *B. pumilus* presented the highest values of protein and fiber content. Moreover, in most of the cases, high values of photosynthetic rate, transpiration rate and stomatal conductance during the cultivation period, resulted in high productivity. Regarding the texture, the size, the sphericity and the ash content of corn grains, it was found that they were not influenced by the application of different treatments of PGPB. The use of certain strains of PGPB, under specific soil and climatic conditions could contribute to better understand which strains are better suited to certain crops.

## Introduction

Plant growth promoting bacteria (PGPB) have been introduced in modern agriculture as a new practice to enhance the growth and the productivity of crops in a sustainable way. Many studies have been conducted to confirm this promising practice^1,2^. PGPB used as biostimulants have been found to enhance the tolerance in abiotic stress of crops, as well as their growth and quality traits^1–3^. Species of bacteria such as *Bacillus* spp., *Pseudomonas* spp. and *Acinetobacter* spp. among others have been used as plant growth promoters^4,5^, these bacteria can be mostly found in the rhizosphere as their natural habitat, but also in aquatic environment and even inside plants as endophytic microorganisms^6–8^.

Plant growth promoting bacteria have a vast variety of mechanisms of action^9^. The production of growth regulators and the change or the release of hormones such as auxin and cytokinins^10,11^ are some of these functions along with the ability to boost the uptake of nutrients by plants^8^ and also increase their abiotic stress tolerance^3,12^. Moreover, some bacteria present the ability to create an antagonistic environment to other phytopathogens^13,14^, solubilize phosphorus^15,16^ and make N_2_ fixation^17,18^. Different strains of PGPB species had been found to affect differently a variety of crops. Studies have revealed that the use of indigenous strains of PGPB are more likely to be better adapted in their environmental conditions and can be more efficient and competitive compared to the non-indigenous strains^19,20^. Moreover, many researchers use endophytic bacteria strains isolated from the roots of plants of the same plant species. In a recent study, Lipkova et al.^21^ isolated three endophytic bacteria strains *Priestia megaterium, Bacillus flexus* and *Bacillus subtilis* from the roots of maize plants and used them as biostimulants to evaluate their effectiveness in maize’s growth.

The increasing research activity of the last years has revealed many other benefits of the use of PGPB, such as stress tolerance and enhancing the plant defense. Specific strains of bacteria have been found that can protect maize plants from salinity damage. For example, it was found that some of the *Azotobacter* strains can mitigate the saline stress^3^. Moreover, the strain SG-5 of *Acinetobacter* sp. can help maize plants tolerate Cd stress by combining the optimal level of K, Ca, Mg, Zn and increased anti-oxidative potential that affected their growth in a positive way^4^. PGPB are not only used for abiotic stress avoidance, but also for enhancing the plant defense for certain pathogens. Cui et al.^14^ found that the strain B9601-Y2 of *Bacillus amyloliquefaciens* can control the southern corn leaf blight by being antagonistic with the phytopathogen *Bipolaris maydis*.

Another important feature to consider in the use of PGPB, is the method of application. A recent study performed at maize, illustrated that foliar and/or ground application of PGPB promoted certain physiological and molecular processes leading to improved growth and productivity of the plants as well as to enhanced quality and nutritional characteristics of the harvested grains^1^. The results showed that soil application of *Priestia megaterium* and a mix of *Azotobacter chroococcum* with *Bacillus subtilis* stand above all other treatments for the yield measurement, while *Bacillus subtilis* presented better results in quality characteristics.

The aim of this study was to assess the effectiveness of ten plant growth promoting bacteria treatments, on sweet corn cultivation under Mediterranean climatic conditions and defined soil physicochemical characteristics. Measurements of plant growth, physiology and yield of sweet corn were conducted, as well as lab analysis for the quality characteristics of grains in order to investigate the effect of the application of these PGPB strains on sweet corn cultivation.

## Materials and methods

### Experimental site and design

**Figure 1:**
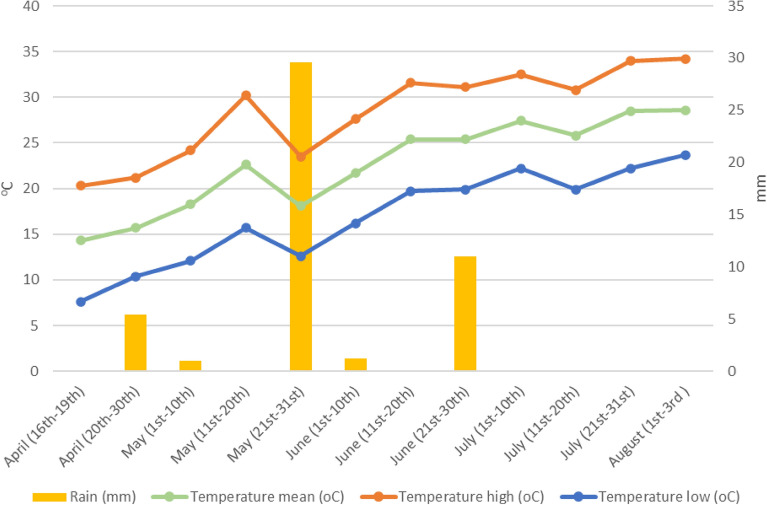
Temperature and precipitation data during the experimental period at Oropos.

The experiment was conducted in the region of Attica at Oropos (38° 18′ N, 23° 45′ E, Altitude 45 m), Greece. Sweet corn hybrid Turbo F1 (Geniki Fytotechniki Athinon, AEVE, Athens, Greece) was sowed on 16 April 2020 and the crop was harvested on 3 August 2020. The temperature and precipitation data of the experimental site during the conduction of the experiment are presented in Fig. 1. This study complies with relevant institutional, national, and international guidelines and legislation.

A completely randomized design with 11 treatments of PGPB was followed. In particular, 9 strains of PGPB were used in which 7 were species of *Bacillus*, 1 species of *Priestia* and 1 species of *Azotobacter* and 1 treatment of a solid Mix consisted of *Priestia megaterium* B004 (3.4 × 10^7^ CFU/cm^3^) + *Azotobacter chroococcum* A004 (1.3 × 10^7^ CFU/cm^3^) at a ratio of mixing 1:2 with neutral pH (6.8–7.2) and zeolite as carrier and 1 control treatment.

Three replications were performed for every treatment and the area of each experimental plot was 6 m^2^. The distance between rows was 75 cm and between plants within the row 20 cm. Each experimental plot consisted of 40 maize plants. The application rate of PGPB was 7 lt/ha for the liquid treatments and 150 kg/ha for the solid Mix treatment.

The PGPB solution was diluted with tap water (1:100) and applied to the soil close to maize plants. Application day was on 26th of May, 40 DAS. All weather conditions (daily mean, high and low temperature and precipitation) during the experiment were retrieved from the NOANN network of the National Observatory of Athens^22^.

Two weeks before sowing a sample was received in the experimental site from four representative points of the field at the depth of 0–30 cm (Table 1). The elements Ca^2+^, Mg^2+^, K^+^ were determined by atomic absorption spectrometry^23^, Zn^2+^, Mn^2+^, Cu^+^ and Fe^3+^were determined by atomic absorption spectrometry using DTPA^24^. Available B was determined using a spectrophotometer, using azomethine-H as the color (yellow) development reagent^25^. Total Nitrogen was determined with ISO, 1995 (11261)^26^, organic matter according to ISO, 1998 (14235)^27^, available Phosphorus with ISO, 1994 (11263)^28^, soil texture was determined using the method of Bouyoucos^29^, the moisture content was determined in a furnace at 105 °C for 24 h and the value of pH was measured with a pH-meter equipped with glass electrode in the saturated paste extract. Total salts were calculated using the results of electrical conductivity and the saturation percentage of the soil samples. Electrical conductivity was determined in an aqueous extract of soil according to ISO 11265:1994^30^.

**Table 1:** Soil physical and chemical properties.

Parameters	Values	Method
Sand (%)	34	Bouyoucos^29^
Silt (%)	28	
Clay (%)	38	
Soil texture	Clay loam	
pH	7.6	pH-meter
Saturation percentage (%)	55	furnace at 105 °C
Electrical conductivity (mS cm^−1^)	1.41	ISO 11265:1994^30^
Total salts (%)	0.05	calculation
Organic matter (%)	4.9	ISO 14235:1998^27^
Total nitrogen (mg g^−1^)	2.2	ISO 11261:1995^26^
Available K (cmoℓ + kg^−1^)	1.2	atomic absorption spectrometry^23^
Available Ca (cmoℓ + kg^−1^)	22	
Available Mg (cmoℓ + kg^−1^)	6.4	
Available P (mg kg^−1^)	87	ISO 11263:1994^28^
Fe-DTPA (mg kg^−1^)	34	DTPA^24^
Cu-DTPA (mg kg^−1^)	3.7	
Zn-DTPA (mg kg^−1^)	8.2	
Mn-DTPA (mg kg^−1^)	15.7	
Available B (mg kg^−1^)	1.5	Bingham^25^

### Cultivation of bacteria

The bacterial strains were collected and belong to Agrounik d.o.o. (Belgrade-Zemun, Serbia). The bacterial strains *Bacillus amyloliquefaciens*, *Bacillus subtilis, Azotobacter chroococcum, Priestia megaterium* belong to the Agrounik collection. These bacteria were isolated from agricultural soil, that was cultivated with maize. *Bacillus mojavensis* and *Bacillus velezensis* were isolated from agricultural soil, that was cultivated with wheat. *Bacillus licheniformis* was also isolated from agricultural soil that was cultivated with rice, and *Bacillus pseudomycoides* from agricultural soil that was cultivated by vegetables. *Bacillus pumilus* was isolated from wastewater from dairy industries. All these bacteria were isolated by the streaking method. Bacterial identification was conducted by sequencing 16 rDNA by the process described by Katsenios et al.^31^. All cultivation was carried out as described previously by Efthimiadou et al.^1^. All bacterial strains were determined by the number of viable cells^32^, pH, production of plant hormone auxin by colourimetric analysis^33^.

The sequence data of the strains with accession numbers have been submitted to GenBank of NCBI database (except *A. chroococcum*). Different colonies were seeded in 100 ml of TSB and *Azotobacter* medium for 24 h, with optical density between 0.3 and 0.35. After this process, 2% of the inoculum was seeded in 3L of the medium. *Bacillus* species were cultivated in Tryptic Soy Broth (TSB) and grown under aerobic conditions at 32 °C with shaking at 200 rpm^34^. *Azotobacter chroococcum* was cultivated in *Azotobacter* medium and grew at 30 °C with shaking at 180 rpm for 72 h. After fermentation the bacteria strains were evaluated for their optimal growth (Colony-forming unit—CFU), pH and production of plant hormone auxin by colourimetric analysis^35^. The bacteria strains that were used in the experiment were *Bacillus amyloliquefaciens* B002 (NCBI: MW562326) with 6.70 pH, 6.5*10^9^ CFU/ml and 38.45 ppm concentration of auxin, *Bacillus licheniformis* B017 (NCBI: MW562833) with 6.15 pH, 6.0*10^9^ CFU/ml and 45.00 ppm concentration of auxin, *Bacillus mojavensis* B010 (NCBI: MW562828) with 5.95 pH, 4.1*10^9^ CFU/ml and 40.52 ppm concentration of auxin, *Bacillus pumilus* W27-4 (NCBI: MW562832) with 6.01 pH, 2.6*10^9^ CFU/ml and 58.10 ppm concentration of auxin, *Bacillus subtilis* Z3 (NCBI: MW396734) with 5.99 pH, 3.0*10^9^ CFU/ml and 43.97 ppm concentration of auxin, *Bacillus pseudomycoides S3* (NCBI: MW687620) with 5.92 pH, 6.0*10^9^ CFU/ml and 39.14 ppm concentration of auxin, *Bacillus velezensis* B006 (NCBI: MW562831) with 6.08 pH, 5.2*10^8^ CFU/ml and 46.03 ppm concentration of auxin, *Azotobacter chroococcum* A004 (NCBI: -) with 7.20 pH, 6.4*10^9^ CFU/ml and 24.00 ppm concentration of auxin, *Priestia megaterium* B004 (NCBI: MW562819) with 6.40 pH, 6.2*10^9^ CFU/ml and 57.76 ppm concentration of auxin and a mix of *Priestia megaterium* B004 (3.4 × 10^7^ CFU/ml) + *Azotobacter chroococcum* A004 (1.3 × 10^7^ CFU/ml) with zeolite as a carrier.

### Measurements

At 70, 84, 98 days after sowing (DAS) dry weight and physiology measurements were performed. Dry weight was measured with a precision balance after the samples (whole plants) were oven-dried at 70 °C for three days. The photosynthetic rate (μmol CO_2_ m^−2^ s^−1^), transpiration rate (mmol H_2_O m^−2^ s^−1^), stomatal conductance (mol m^−2^ s^−1^) of plants were measured with a LCi Leaf Chamber Analysis System (ADC, Bioscientific, Hoddesdon, UK), The LCi Leaf Chamber Analysis System measures these parameters in the field at midday hours with a clear sky, on fully expanded leaves and share the results on the spot. Chlorophyll content (μg/cm^2^), was measured with a portable chlorophyll meter (SPAD), on fully expanded leaves.

### Quality characteristics of harvested corn grains

Τhe harvested corn grains were dried in the shade according to the typical farming practices. The moisture content of the collected corn grains was approximately 10.32 ± 0.06%. Size and sphericity of corn grains were determined according to the method described by Efthimiadou et al.^1^. The color parameters of the corn grains were measured using Minolta Colorimeter (CR-300, Minolta Company, Chuo-Ku, Osaka, Japan) using the CIELAB color space where the L value represents the lightness, the a value the red-green direction of the color and the b the yellow-blue direction. L value indicates the brightness of the product where 0–100 represents dark to light. The a value represents the redness and greenness of the product. A positive value represents more red color. The b value represents the yellow-blue color. A positive b value shows more yellow color. The chroma (C) was also determined according to the Eq. (1)

1 $$Chroma \left(C\right)=\sqrt{\left({a}^{2}\right)+({b}^{2})}$$

The texture analysis was performed by HD-Plus texture analyzer (Stable Micro Systems Ltd., UK) and the Texture Expert Exceed Software for the data analysis. The determination of the textural characteristics of corn grains was performed by a puncture probe of 5 mm diameter. Probe speeds of 1 mm/s during the test, 2 mm/s for pre-test and 10 mm/s for post-test were used throughout the study. All the measurements were performed at ambient conditions and the hardness of the corn seeds was determined and expressed at N.

Quality parameters including moisture, ash, total protein, and total crude fiber content of corn flours were also determined. Ash and crude fiber content of corn flours were determined according to AOAC Official Method 923.03 and 984.04 (Weende Method), respectively. Total protein content analysis of corn flours was conducted by applying the Kjeldahl method (IDF 2008), using a Kjeldahl rapid distillation unit (Protein Nitrogen Distiller DNP-1500-MP, RAYPA, Spain).

### Statistical analysis

A one-way analysis of variance (ANOVA) was performed to evaluate the effect of PGPB application. IBM SPSS software ver. 24 (IBM Corp., Armonk, N.Y., USA) was used to analyze the experimental data. Tukey Honestly Significant Difference (HSD) test at the 5% level of significance (*p* ≤ 0.05) was used for the comparisons of means. In order to examine the predictive significance of this dataset, Python 3.7, the Scikit Learn library and the Pinguin library were used, testing thirteen different algorithms in tenfold cross validation experiments. In total over 15,000 different models were tested and estimated. The database that we used for saving the data was MongoDB, a NoSQL database, which is based on JSON format.

## Results

### Plant growth

**Figure 2:**
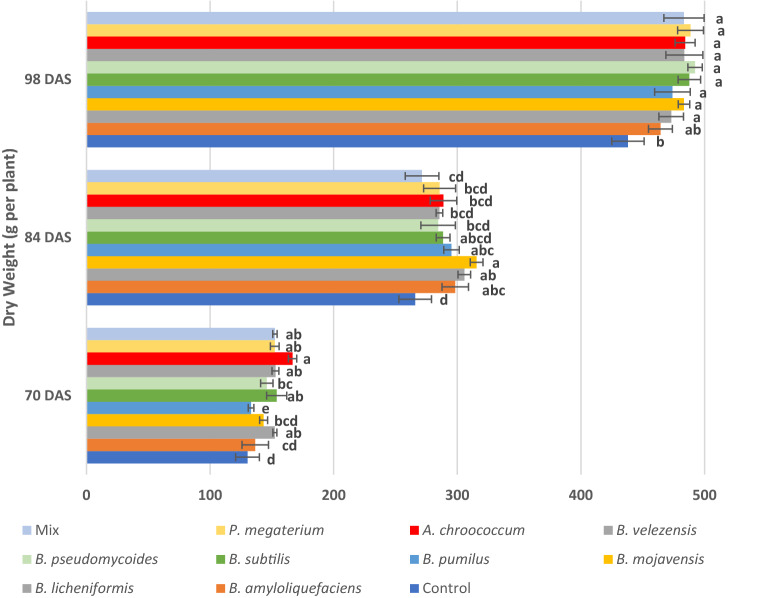
Effect of PGPB on dry weight of whole plants 70, 84 and 98 DAS. DAS, days after sowing. Mix: Mix of *Priestia megaterium* + *Azotobacter chroococcum*. Means followed by the same letter for treatments are not significantly different according to Tukey Honestly Significant Difference (HSD) test (*p* < 0.05). Values presented are mean values of three replicates ± standard deviation. F values of ANOVA: 12.497***, 6.107*** and 5.670*** for 70 84 and 98 DAS respectively. Significance level: ***p < 0.001.

At the first measurement (70 DAS) of dry weight, *A. chroococcum* treatment performed the best (166.7 g per plant) among the other treatments with statistically significant differences compared to control (Fig. 2). However, at the second measurement (84 DAS), *B. mojavensis, B. licheniformis* and *B. amyloliquefaciens* treatments (315.6 g, 305.7 g and 298.3 g respectively) were the highest values compared to all the other treatments. At the final measurement (98 DAS) most of the PGPB treatments had no statistically significant differences among them, but they were significantly higher than the control. In particular, *B. pseudomycoides* (492.3 g), *P. megaterium* (488.7 g) and *B. subtilis* (487.7 g) gave the highest values of dry weight.

### Physiology measurements

In the case of photosynthetic rate (Table 2), at the first measurement (70 DAS), *B. velezensis* (42.3 μmol CO_2_ m^−2^ s^−1^) and *B. pumilus* (41.8 μmol CO_2_ m^−2^ s^−1^) and *B. pseudomycoides* (41.6 μmol CO_2_ m^−2^ s^−1^) gave the highest values that differ statistical significantly compared to the treatments of *P. megaterium*, Control and Mix (38, 37.5 and 37.4 μmol CO_2_ m^−2^ s^−1^ respectively). At the second measurement (84 DAS) the treatment of *B. pumilus* (43.6 CO_2_ m^−2^ s^−1^) gave the highest value of photosynthetic rate with statistically significant differences compared to the other treatments, except for *B. subtilis*, *B. pseudomycoides* and *B. velezensis*. However, at the last measurement (98 DAS) it was *B. licheniformis* treatment (37 μmol CO_2_ m^−2^ s^−1^) that kept its photosynthetic rate in high values whereas the other treatments had lower values.

**Table 2:** Effect of PGPB on photosynthetic rate of maize plants 70, 84 and 98 DAS.

Treatment	Photosynthetic rate (μmol CO_2_ m^−2^ s^−1^)		
	70 DAS	84 DAS	98 DAS
Control	37.5 ± 1.2^cd^	37.5 ± 0.6^e^	32.9 ± 0.4^c^
*B. amyloliquefaciens*	39.8 ± 1.1^abcd^	38.3 ± 0.3^de^	33.8 ± 0.8^bc^
*B. licheniformis*	40.9 ± 1.3^ab^	39.2 ± 0.6^de^	37.0 ± 0.5^a^
*B. mojavensis*	39.6 ± 1.0^abcd^	41.5 ± 0.3^bc^	35.6 ± 1.0^ab^
*B. pumilus*	41.8 ± 1.9^a^	43.6 ± 0.6^a^	34.1 ± 0.8^bc^
*B. subtilis*	40.6 ± 0.2^abcd^	41.9 ± 0.3^ab^	35.3 ± 0.6^ab^
*B. pseudomycoides*	41.6 ± 1.2^a^	42.7 ± 1.0^ab^	34.9 ± 0.3^b^
*B. velezensis*	42.3 ± 1.0^a^	42.0 ± 0.7^ab^	35.5 ± 0.9^ab^
*A. chroococcum*	40.7 ± 0.8^abc^	41.2 ± 0.6^bc^	34.6 ± 0.3^bc^
*P. megaterium*	38.0 ± 0.4^bcd^	40.0 ± 0.6^cd^	34.6 ± 0.0^bc^
Mix	37.4 ± 1.1^d^	40.0 ± 0.6^cd^	35.3 ± 0.5^ab^
F_treat_	7.315***	30.250***	8.778***

DAS, days after sowing. Mix: Mix of *Priestia megaterium* B004 + *Azotobacter chroococcum* A004 with zeolite as a carrier. Means followed by the same letter for treatments are not significantly different according to Duncan test (*p* < 0.05). Values presented are mean values of three replicates ± standard deviation. Significance levels: ***p < 0.001.

Concerning transpiration rate, at the first measurement (70 DAS), all PGPB treatments presented statistically significant higher values compared to control (Table 3). At the second measurement (84 DAS), however, *A. chroococcum* (7.61 mmol H_2_O m^−2^ s^−1^) was the treatment with the highest value followed by *B. subtilis* (7.52 mmol H_2_O m^−2^ s^−1^) and *B. pumilus* (7.40 mmol H_2_O m^−2^ s^−1^) with statistically significantly differences compared to control and the other PGPB treatments except for *B. mojavensis* and *B. pseudomycoides*. The latter was the one with the highest value in the last measurement with 6.52 mmol H_2_O m^−2^ s^−1^ with no statistically significant differences from *B. subtilis* (6.32 mmol H_2_O m^−2^ s^−1^) and *B. pumilus* (6.10 mmol H_2_O m^−2^ s^−1^) treatments that followed in high values.

**Table 3:** Effect of PGPB on transpiration rate, of maize plants 70, 84 and 98 DAS.

Treatment	Transpiration rate (mmol H_2_O m^−2^ s^−1^)		
	70 DAS	84 DAS	98 DAS
Control	6.46 ± 0.04^b^	6.41 ± 0.15^cd^	5.42 ± 0.21^c^
*B. amyloliquefaciens*	7.27 ± 0.45^a^	6.32 ± 0.05^d^	5.54 ± 0.29^c^
*B. licheniformis*	7.36 ± 0.48^a^	6.98 ± 0.16^b^	5.43 ± 0.33^c^
*B. mojavensis*	7.19 ± 0.22^ab^	7.32 ± 0.19^ab^	5.78 ± 0.19^bc^
*B. pumilus*	7.32 ± 0.21^a^	7.40 ± 0.20^ab^	6.10 ± 0.29^abc^
*B. subtilis*	7.32 ± 0.10^a^	7.52 ± 0.33^a^	6.32 ± 0.14^ab^
*B. pseudomycoides*	7.45 ± 0.08^a^	7.27 ± 0.24^ab^	6.52 ± 0.27^a^
*B. velezensis*	6.87 ± 0.15^ab^	7.20 ± 0.16^ab^	5.73 ± 0.32^bc^
*A. chroococcum*	7.37 ± 0.26^a^	7.61 ± 0.20^a^	5.97 ± 0.17^abc^
*P. megaterium*	7.22 ± 0.21^ab^	6.89 ± 0.11^bc^	5.97 ± 0.12^abc^
Mix	7.15 ± 0.32^ab^	6.97 ± 0.11^b^	6.06 ± 0.30^abc^
F_treat_	3.416**	15.256***	6.061***

DAS, days after sowing. Mix: Mix of *Priestia megaterium* B004 + *Azotobacter chroococcum* A004 with zeolite as a carrier. Means followed by the same letter for treatments are not significantly different according to Duncan test (*p* < 0.05). Values presented are mean values of three replicates ± standard deviation. Significance levels: ***p < 0.001, **p < 0.01.

Stomatal conductance of plants (Table 4) treated with *B. licheniformis* (0.60 mol m^−2^ s^−1^), *B. pseudomycoides* (0.59 mol m^−2^ s^−1^), as well as *B. subtilis* (0.57 mol m^−2^ s^−1^), were statistically significantly higher than the treatments of control, *A. chroococcum*, *P. megaterium* and Mix at the first measurement (70 DAS). At the second measurement (84 DAS) *B. velezensis* (0.53 mol m^−2^ s^−1^) followed by *B. subtilis* (0.51 mol m^−2^ s^−1^) *B. pseudomycoides* (0.51 mol m^−2^ s^−1^) *A. chroococcum* (0.48 mol m^−2^ s^−1^) and *B. velezensis* (0.48 mol m^−2^ s^−1^) were the treatments with statistically significantly higher values compared to control and the rest of the PGPB. At the third measurement (98 DAS), *B. velezensis* (0.45 mol m^−2^ s^−1^) was still the treatment with the highest value, again followed by *B. subtilis* (0.43 mol m^−2^ s^−1^) and *B. pseudomycoides* (0.43 mol m^−2^ s^−1^) with statistically significant differences compared to control.

**Table 4:** Effect of PGPB on stomatal conductance, of maize plants 70, 84 and 98 DAS.

Treatment	Stomatal conductance (mol m^−2^ s^−1^)		
	70 DAS	84 DAS	98 DAS
Control	0.48 ± 0.03^bcd^	0.44 ± 0.01^cd^	0.40 ± 0.01^bcd^
*B. amyloliquefaciens*	0.51 ± 0.03^abcd^	0.43 ± 0.02^d^	0.39 ± 0.01^d^
*B. licheniformis*	0.60 ± 0.01^a^	0.45 ± 0.03^cd^	0.39 ± 0.01^cd^
*B. mojavensis*	0.51 ± 0.03^abcd^	0.48 ± 0.01^abc^	0.42 ± 0.0^abcd^
*B. pumilus*	0.50 ± 0.03^abcd^	0.47 ± 0.02^bcd^	0.41 ± 0.01^bcd^
*B. subtilis*	0.57 ± 0.05^abc^	0.51 ± 0.02^ab^	0.43 ± 0.01^ab^
*B. pseudomycoides*	0.59 ± 0.07^ab^	0.51 ± 0.02^ab^	0.43 ± 0.01^abc^
*B. velezensis*	0.51 ± 0.05^abcd^	0.53 ± 0.02^a^	0.45 ± 0.02^a^
*A. chroococcum*	0.46 ± 0.05^cd^	0.48 ± 0.01^abc^	0.40 ± 0.01^bcd^
*P. megaterium*	0.46 ± 0.04^cd^	0.47 ± 0.02^bcd^	0.42 ± 0.02^abcd^
Mix	0.43 ± 0.01^d^	0.47 ± 0.02^bcd^	0.42 ± 0.01^abcd^
F_treat_	5.736***	9.222***	6.471***

DAS, days after sowing. Mix: Mix of *Priestia megaterium* B004 + *Azotobacter chroococcum* A004 with zeolite as a carrier. Means followed by the same letter for treatments are not significantly different according to Duncan test (*p* < 0.05). Values presented are mean values of three replicates ± standard deviation. Significance levels: ***p < 0.001.

Chlorophyll content measurements at 70 DAS, showed that the treatment of *B. mojavensis* (61.4 μg/cm^2^) had the highest value with no statistically significant differences from *B. licheniformis, B. pseudomycoides*, *B. subtilis*, *B. velezensis* and Mix, but statistically significantly higher than the control and the other PGPB treatments (Table 5). At the second measurement (84 DAS) all PGPB treatments gave statistically significant higher values than the control except for *B. amyloliquefaciens* and *B. licheniformis* that in both measurements had no statistically significant differences compared to control. At the third measurement (98 DAS), the treatments of *B. pumilus*, *B. subtilis*, *B. pseudomycoides*, *B. velezensis* and *P. megaterium* gave the highest values with statistically significant differences compared to control.

**Table 5:** Effect of PGPB on chlorophyll content, of maize plants 70, 84 and 98 DAS.

Treatment	Chlorophyll content (μg/cm^2^)		
	70 DAS	84 DAS	98 DAS
Control	44.8 ± 1.5^e^	46.5 ± 1.1^c^	37.7 ± 1.0^c^
*B. amyloliquefaciens*	51.8 ± 1.9^bcde^	48.4 ± 0.7^bc^	38.3 ± 0.6^bc^
*B. licheniformis*	60.1 ± 1.9^ab^	49.1 ± 0.6^abc^	38.5 ± 1.0^bc^
*B. mojavensis*	61.4 ± 0.9^a^	53.5 ± 2.5^ab^	40.4 ± 1.4^abc^
*B. pumilus*	55.7 ± 4.4^abc^	53.0 ± 2.9^ab^	41.7 ± 2.3^ab^
*B. subtilis*	57.5 ± 6.2^ab^	53.2 ± 1.8^ab^	41.7 ± 0.7^ab^
*B. pseudomycoides*	58.1 ± 2.3^ab^	53.0 ± 2.7^ab^	43.2 ± 2.0^a^
*B. velezensis*	53.7 ± 3.1^abcd^	54.5 ± 0.9^a^	42.2 ± 1.3^ab^
*A. chroococcum*	47.9 ± 1.6^cde^	52.1 ± 1.9^ab^	41.5 ± 0.5^abc^
*P. megaterium*	46.7 ± 1.8^de^	53.4 ± 2.7^ab^	41.8 ± 1.7^ab^
Mix	53.2 ± 3.5^abcde^	52.7 ± 1.5^ab^	40.6 ± 0.9^abc^
F_treat_	9.845***	5.358**	5.420***

DAS, days after sowing. Mix: Mix of *Priestia megaterium* B004 + *Azotobacter chroococcum* A004 with zeolite as a carrier. Means followed by the same letter for treatments are not significantly different according to Duncan test (*p* < 0.05). Values presented are mean values of three replicates ± standard deviation. Significance levels: *** *p* < 0.001, ** *p* < 0.01.

### Yield

**Figure 3:**
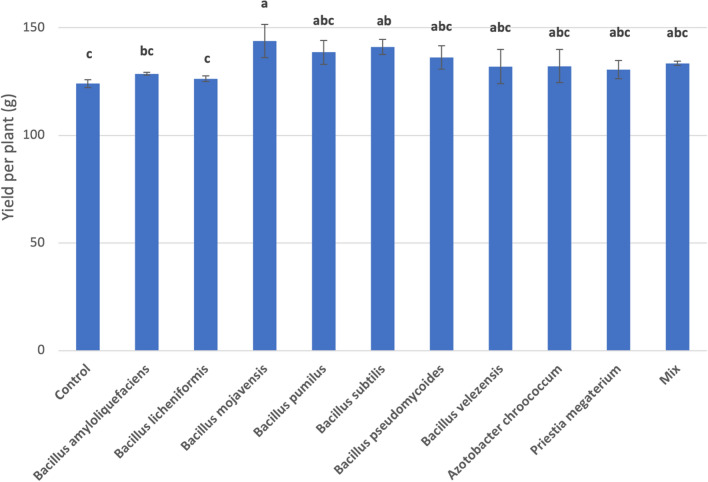
Effect of PGPB on yield per plant. Mix: Mix of *Priestia megaterium* + *Azotobacter chroococcum*. Means followed by the same letter for treatments are not significantly different according to Tukey Honestly Significant Difference (HSD) test (*p* < 0.05). Values presented are mean values of three replicates ± standard deviation. F value of ANOVA: 4.446**. Significance level: **p < 0.01.

Plant growth promoting bacteria that were applied on the soil of maize plants resulted in yield increase for all the tested treatments. The highest yield was presented by *B. mojavensis* (144 g per plant) followed by *B. subtilis* (141.2 g per plant), with statistically significant differences compared to control (Fig. 3). Even though all the values of the PGPB treatments were higher than the control, the applications with Mix (133.6 g per plant), *A. chroococcum* (132.2 g per plant)*, B. velezensis* (132 g per plant)*, P. megaterium* (130.6 g per plant)*, B. amyloliquefaciens* (128.7 g per plant) and *B. licheniformis* (126.4 g per plant) strains had no statistically significant differences among them or compared to the control (124.1 g per plant).

### Quality characteristics of harvested corn grains

The effect of different PGPB on the quality characteristics (color parameters, texture, size, sphericity, ash, protein and fiber content) of the harvested corn grains is presented in Tables 6, 7 and 8. Concerning the color of corn grains, their lightness (L) ranged from 66.04 to 73.63, the yellow-blue index b ranged from 35.60 to 42.69, the red index a ranged from 1.78 to 5.08 and the chroma parameter ranged from 35.69 to 44.45. The application of different PGPB significantly influenced the performance of corn grains (*p* < 0.05); except for the interaction on the red-green index, L, b and chroma parameters were statistically significantly different between the different PGPB treatments. The highest values of L color parameter of corn grains were obtained from the treatment of *B. licheniformis* (71.21)*,* followed by *B. pseudomycoides* (69.73) and *P. megaterium* (68.65)*.* The highest values of b index and chroma parameter of corn grains were obtained from the treatment of Mix (Mix of *P. megaterium* + *Azotobacter chroococcum*) and the lowest values from the treatment of *B. licheniformis* (Table 6).

**Table 6:** Effect of PGPB on color parameters of sweet corn grains.

Treatment	L	a	b	Chroma
Control	65.97 ± 0.72^c^	3.40 ± 0.43	41.14 ± 0.78^ab^	41.28 ± 0.77^ab^
*B. amyloliquefaciens*	71.21 ± 2.19^a^	3.83 ± 0.53	39.87 ± 0.03^b^	40.05 ± 0.07^b^
*B. licheniformis*	66.31 ± 0.07^bc^	2.16 ± 0.40	37.69 ± 0.66^b^	37.76 ± 0.66^b^
*B. mojavensis*	67.89 ± 0.62^abc^	2.92 ± 1.41	40.77 ± 1.38^ab^	40.89 ± 1.41^ab^
*B. pumilus*	67.55 ± 1.25^bc^	3.00 ± 0.96	40.70 ± 0.35^ab^	40.82 ± 0.36^ab^
*B. subtilis*	68.87 ± 1.41^abc^	3.73 ± 1.24	38.97 ± 0.73^b^	39.16 ± 0.84^b^
*B. pseudomycoides*	69.73 ± 1.97^ab^	3.21 ± 0.43	38.56 ± 0.37^b^	38.69 ± 0.40^b^
*B. velezensis*	67.46 ± 0.55^bc^	2.62 ± 0.95	39.37 ± 1.58^b^	39.46 ± 1.58^b^
*A. chroococcum*	67.84 ± 0.62^abc^	4.11 ± 0.44	40.00 ± 0.68^b^	40.21 ± 0.64^ab^
*P. megaterium*	68.65 ± 0.72^abc^	3.81 ± 1.25	39.88 ± 3.83^b^	40.07 ± 3.93^b^
Mix	67.50 ± 0.25^bc^	3.05 ± 0.67	44.27 ± 0.12^a^	44.37 ± 0.11^a^
F_treat_	4.710**	1.350^ns^	4.528**	4.331**

Mix: Mix of *Priestia megaterium* B004 + *Azotobacter chroococcum* A004 with zeolite as a carrier. Values presented are mean values of three replicates ± standard deviation. Means followed by the same letter for treatments are not significantly different according to Tukey Honestly Significant Difference (HSD) test (*p* < 0.05). Significance levels: ** *p* < 0.01; *** *p* < 0.001; ns: not significant.

The texture, the size and the sphericity of the harvested corn grains varied between 12.67–24.88 N, 6.76–7.14 mm and 0.48–0.55, respectively, across the different treatments of PGPB (Table 7). Regarding the ash content of corn grains across all the treatments, its values ranged from 1.19 to 2.77% (Table 8). However, it was found that the texture, the size, the sphericity and the ash content of corn grains were not statistically influenced by the application of different treatments of PGPB.

**Table 7:** Effect of PGPB on texture, size and sphericity of sweet corn grains.

Treatment	Texture (N)	Size (mm)	Sphericity
Control	16.83 ± 2.21	6.99 ± 0.09	0.52 ± 0.02
*B. amyloliquefaciens*	20.40 ± 1.50	7.05 ± 0.08	0.54 ± 0.01
*B. licheniformis*	18.12 ± 0.55	6.99 ± 0.14	0.51 ± 0.02
*B. mojavensis*	18.08 ± 1.01	7.04 ± 0.07	0.52 ± 0.01
*B. pumilus*	18.30 ± 2.54	6.85 ± 0.33	0.49 ± 0.01
*B. subtilis*	19.07 ± 1.47	7.15 ± 0.21	0.52 ± 0.01
*B. pseudomycoides*	19.94 ± 4.37	7.08 ± 0.07	0.53 ± 0.01
*B. velezensis*	17.42 ± 4.18	7.03 ± 0.25	0.52 ± 0.02
*A. chroococcum*	18.16 ± 2.48	7.02 ± 0.16	0.51 ± 0.01
*P. megaterium*	18.75 ± 0.88	6.97 ± 0.05	0.51 ± 0.01
Mix	16.75 ± 0.95	7.15 ± 0.20	0.52 ± 0.02
F_treat_	0.712^ns^	0.720^ns^	1.962^ns^

Mix: Mix of *Priestia megaterium* B004 + *Azotobacter chroococcum* A004 with zeolite as a carrier. Values presented are mean values of three replicates ± standard deviation. Means followed by the same letter for treatments are not significantly different according to Tukey Honestly Significant Difference (HSD) test (*p* < 0.05). Significance levels: ns: not significant.

**Table 8:** Effect of PGPB on ash, protein, crude fiber content of sweet corn grains.

Treatment	Ash (%)	Proteins (%)	Fibers (%)
Control	2.40 ± 0.12	11.97 ± 0.26^d^	2.66 ± 0.22^c^
*B. amyloliquefaciens*	2.36 ± 0.54	14.94 ± 0.78^abc^	3.49 ± 0.34^bc^
*B. licheniformis*	2.56 ± 0.13	15.05 ± 0.89^abc^	3.43 ± 0.13^bc^
*B. mojavensis*	2.31 ± 0.15	17.12 ± 0.77^a^	4.54 ± 0.70^ab^
*B. pumilus*	2.53 ± 0.30	16.45 ± 0.21^a^	5.20 ± 0.72^a^
*B. subtilis*	2.66 ± 0.05	15.62 ± 0.45^ab^	4.95 ± 0.59^a^
*B. pseudomycoides*	2.08 ± 0.05	12.83 ± 0.81^cd^	3.76 ± 0.03^bc^
*B. velezensis*	2.04 ± 0.74	14.98 ± 1.19^abc^	3.58 ± 0.16^bc^
*A. chroococcum*	2.33 ± 0.06	13.49 ± 0.43^bcd^	3.69 ± 0.30^bc^
*P. megaterium*	2.58 ± 0.02	13.36 ± 0.56^bcd^	3.54 ± 0.39^bc^
Mix	2.45 ± 0.13	15.28 ± 1.60^ab^	3.02 ± 0.14^c^
F_treat_	1.278^ns^	10.933***	11.052***

Mix: Mix of *Priestia megaterium* B004 + *Azotobacter chroococcum* A004 with zeolite as a carrier. Values presented are mean values of three replicates ± standard deviation. Means followed by the same letter for treatments are not significantly different according to Tukey Honestly Significant Difference (HSD) test (*p* < 0.05). Significance levels: ***p < 0.001; ns: not significant.

As far as the protein content is concerned, the different treatments highly influenced the protein content of corn grains (*p* < 0.001) (Table 8). The protein content across all PGPB treatments varied from 11.68 to 17.66%. The highest protein content with statistically significant differences compared to control was found in corn grains obtained by *B. mojavensis* application (17.12%), followed by *B. pumilus* (16.45%) and *B. subtilis* (15.62%), Mix (15.28%), *B. licheniformis* (15.02%), *B. velezensis* (14.98%) and *B. amyloliquefeciens* (14.94%) with no statistically significant differences among them. In particular, the protein content of corn grains was approximately 43%, 37% and 30% higher in *B. mojavensis, B. pumilus* and *B. subtilis* treatments, respectively, compared to the control.

The fiber content of corn grains was also significantly influenced by different PGPB treatments (*p* < 0.001) (Table 8). The fiber content across all treatments ranged from 2.41 to 5.62%. The highest fiber content was found in corn grains obtained by *B. pumilus* (5.20%), *B. subtilis* (4.95%) and *B. mojavensis* (4.54%) application. In particular, the fiber content of corn grains was approximately 95%, 86% and 71% higher in *B. pumilus*, *B. subtilis* and *B. mojavensis*, treatments, respectively, compared to the control.

The application of PGPB and especially *B. pumilus*, *B. subtilis* and *B. mojavensis* resulted in corn grains with improved quality characteristics in terms of total protein and crude fiber content without affecting their physical characteristics (texture, size, sphericity) could be a desirable trait for the food industry.

### Feature coefficiency using machine learning models

In order to find which plant growth and physiology measurements appear to be correlated with the yield, protein, fiber and texture of sweet corn, 12 different machine learning algorithms were tested, in 9 different metrics which are used to determine the best model in terms of efficiency.

**Figure 4:**
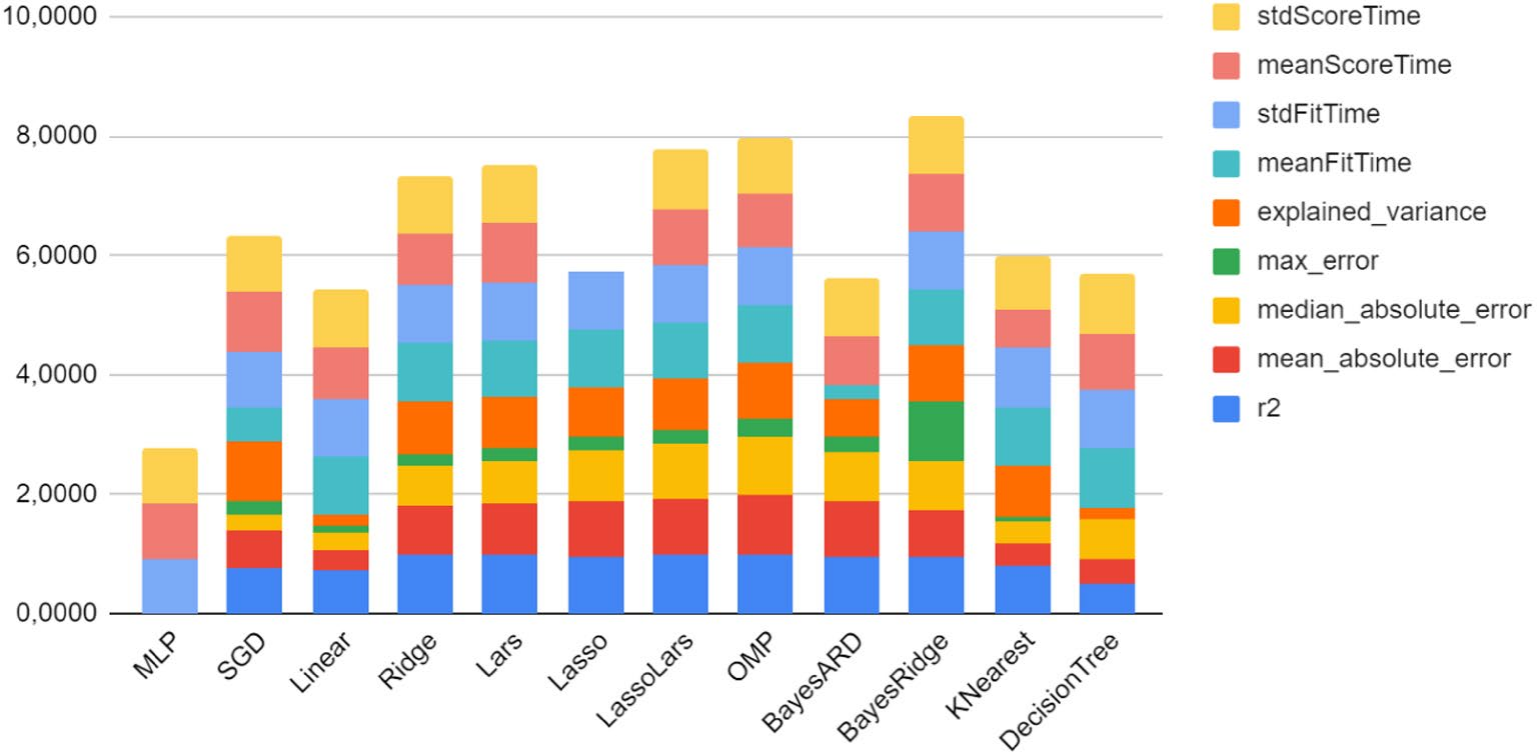
Comparison of normalized error metrics and STDs of the 12 algorithms used.

As we can see in Fig. 4, the Bayesian Ridge algorithm has the best results in terms of speed and efficiency. Using this algorithm, we extracted the feature importance of the variables. This will help us allocate which of the field measurements are highly correlated with the yield, protein, fiber and texture of sweet corn. The model consists of five different measurements taken in three different timestamps (70 days, 84 days and 98 days).

**Figure 5:**
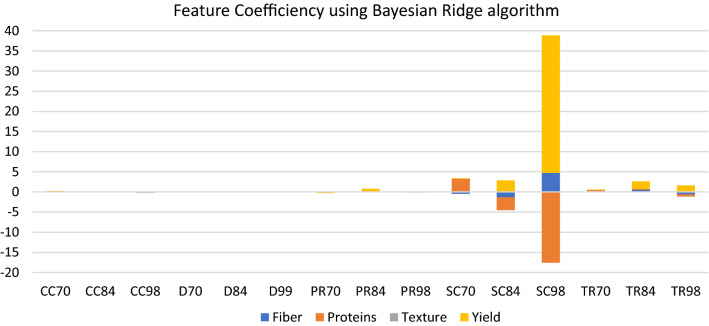
Feature coefficiency of the Bayesian Ridge Algorithms. CC, chlorophyll content; D, dry weight; PR, photosynthetic rate; SC, stomatal conductance; TR, transpiration rate.

Since the Bayes Ridge algorithm is based on the linear regression we used the feature coefficiency in order to find correlations. The feature coefficiency represents the distribution of the weights to the features, as deduced by the algorithm on its formulated function. Each feature consists of the measurements for all the treatments and replications. Positive values show that the measurements are important for the model, while negative values show that the measurements are inversely proportional for the predicted value of the model. In Fig. 5, the feature coefficiency using the Bayes Ridge algorithm was extracted. As we can see the SC 98 metric is also the most important measurement for the yield as well as for the fiber in the model, while SC70 is the most important measurement for the proteins. In addition, SC 98 seems to be inversely proportional for the proteins. Moreover, there are no measurements that were correlated to the texture of the maize.

## Discussion

Various bacteria strains have been used over the years to increase maize yield. Martins et al.^36^ used *Azospirillum brasilense* strain Sp245, *A. brasilense* strains AbV5 + AbV6, *Herbaspirillum seropedicae* strain ZAE94 and found that these strains can increase maize yield with and without N fertlization, however it is very interesting that the protein content was not affected by the PGPB. Sandini et al.^37^ tested *Pseudomonas fluorescens* via seed inoculation in maize along with N fertilization and their results showed that the PGPB not only increased maize’s biomass accumulation but also the grain yield, significantly different from the control. Moreover, Eliaspour et al.^38^ recorded that inoculation with a combination of strain *Pseudomonas putida* 146 and mycorrhiza *Glomus imoseaea* enhanced maize’s yield, chlorophyll content and 1000-seed weight.

Recently the researchers have focused their efforts to evaluate the effect of certain strains of PGPB on a variety of crops. For instance, de Aquino et al.^39^ used 40 PGPB isolates to assess their effect on maize and sorghum growth. Among those isolates *B. subtilis, B. pumilus* and *B. megaterium* (*P. megaterium*) can be found. Most of isolates of these genera showed a height, shoot dry weight and chlorophyll content increase compared to the non-fertilized control. That comes in agreement with our findings because the strains of *B. subtilis, B. pumilus* and *P. megaterium* also presented significantly higher values of maize dry weight and chlorophyll content. Other strains of *B. subtilis, B. pumilus* and *B. megaterium* (*P. megaterium*) had been also found to have a positively effect on chlorophyll content^40^. The chlorophyll content seems to be positively affected by various strains of these genera and thus raising the question if this is a genera characteristic effect on maize plants. Efthimiadou et al.^1^ found that *A. chroococcum* strain B002 increased maize dry weight. Our results confirm these measurements as once again *A. chroococcum* strain B002 presented the highest value of dry weight. Also the crude fiber content findings are in accordance with the study of Efthimiadou et al.^1^, who reported that *B. subtilis* treatment resulted in corn grains with the highest value of crude fiber content compared to the other PGPB (*Azotobacter chroococcum, Bacillus megatherium* and their mixes) treatments and the untreated grains. Moreover, *A. chroococcum* strains have been found to also increase cotton growth^41^ and the yield of spring wheat^42^. In a recent study, Li et al.^43^ used the strain MGW9 of *Bacillus* sp. at maize seeds by biopriming, their results showed that this use improved significantly not only the chlorophyl content of maize plants but also the dry weight of the plant. This comes in agreement with our results, that showed *Bacillus* genera presented high values in both parameters and it seems that they have a positive effect in maize growth.

In case of texture, size and sphericity of maize our results are in agreement with Efthimiadou et al.^1^ that reported no significant differences between texture as well as size and sphericity of maize treated by *Azotobacter chroococcum, Bacillus subtilis, Bacillus megatherium* and their mixes, respectively. In 2021, Katsenios et al.^31^ used these bacteria strains to evaluate their effect in the growth, yield and quality characteristics of tomato plants. The results presented in this study showed that the same strains of PGPB affect those two crops differently, however, is seems that some strains like *Bacillus subtilis* Z3 and *Priesta megaterium* B004 have a positive effect in plants growth and yield. The results of such studies contribute to better understand which strains are better suited to various crops.

The use of Machine Learning models allows us to use computational power in order to extract important data^44^. Feature coefficiency has proven to be important in several fields of study such as agriculture or medicine^45,46^. Important relations for the feature that we study can be extracted allowing us to understand more about the cultivation and the environmental conditions. The use of this state-of-the-art technique is growing, since it can be used as a tool for finding important measurements at the field, as well as for finding out the best algorithm for this type of data^47^.

Novel mining approaches can be used to improve multivariate based methods. Specifically, agricultural measurements, such as yield, can be affected by a variety of features, which can be extracted by different measurements. In a recent study concerning maize yield, a machine learning model based on the K-means algorithm was used for the correlation of the features^48^. In a similar study, a dataset of 598 features was used in order to find the most important one in corn yield. Eight algorithms were tested using 4 different metrics. Random Forest had the best average score^49^. Moreover, 3 different algorithms were used in a dataset of 45 different features aiming at finding the important ones for yield. Random Forest algorithm had the best score in the 5 metrics used^50^. Algorithms can find the most important features and combine the features that contribute to high yield or to features of high importance. Agricultural data, combined with algorithms, can be analysed even if they are complex and they do not follow the same distribution pattern^51^. Moreover, such technologies have been used in the past in order to find the measurements that affect the phenotype. Eleven phenotypic parameters were used in order to find correlation between the measurements in maize cultivation^52^. In another study 14 different measurements were used as an input in order to determine the phenotypic correlation between yield and other traits^53^. Finally, path coefficient analysis (PCA) has been used in order to determine both quality characteristics of maize, such as protein, and phenotypic attributes^54,55^. In research about important features, regarding sugarcane yield, 11 algorithms were tested in a dataset of 32 features. Random Forest algorithm had the best results. Air temperature was the most important feature^56^. Lastly, four different algorithms were tested in order to find the most important feature in blueberry yield. The best model was Multiple Linear Regression and it proved that the density of Bumblebees is the most important feature^57^. This technique can also be applied in other data types, such as soil or water. In research about soil consolidation, 12 different soil features were used. In order to find the best algorithms, four different models were tested. Results showed that Lasso achieved the best performance in and the importance of the features was extracted using this algorithm^58^. In another experiment about soil, 3 different algorithms were tested in their ability to predict soil drying, based on soil and weather data. K-nearest neighbours (KNN) had the best results in finding the importance of each feature^59^.

## Conclusions

The use of ten treatments of PGPB applied at the soil of sweet corn cultivation, affected the plant growth and physiology measurements, as well as the quantity and the quality of the harvested production. The results of the very important measurement of the quantity of production, indicated that *B. mojavensis* increased yield by 16%, *B. subtilis* by 13.8%, *B. pumilus* by 11.8% and *B. pseudomycoides* by 9.8% compared to control. In addition, the harvested grains of the plants treated with *B. mojavensis*, *B. subtilis* and *B. pumilus* presented the highest values of protein and fiber content. The increased dry weight of all PGPB treatments, in combination with the high values of the chlorophyll content during the cultivation period, resulted in enhanced yield. Regarding physiology measurements, in most of the cases, high values of photosynthetic rate, transpiration rate and stomatal conductance during the cultivation period, resulted in high productivity. An interesting finding was that *B. mojavensis* although it presented more moderate values for the physiology measurements, finally gave the highest yield and protein content.

This study contributes to better understand which strains are better suited to certain crops. The performance of each of the examined PGPB, could be attributed to the soil and climatic conditions of the experimental field. Further research is required to determine, the strain and the species of the PGPB, the quantity and the time of application for different crops.
